# Senolytic effect of high intensity interval exercise on human skeletal muscle

**DOI:** 10.18632/aging.204511

**Published:** 2023-02-08

**Authors:** Wei-Horng Jean, Yu-Wen Hsieh, Li-Fan Lai, Luthfia Dewi, Yu-Chieh Liao, Mengxin Ye, Szu-Hsien Yu, Chung-Lan Kao, Chih-Yang Huang, Chia-Hua Kuo

**Affiliations:** 1 Department of Anesthesiology, Far East Memorial Hospital, New Taipei City 220, Taiwan, ROC; 2 Laboratory of Exercise Biochemistry, University of Taipei, Taipei City 11153, Taiwan, ROC; 3 Department of Leisure Industry and Health Promotion, National Ilan University, Ilan, Taiwan, ROC; 4 Department of Physical Medicine and Rehabilitation, Taipei Veterans General Hospital, Taipei City, Taiwan, ROC; 5 Cardiovascular and Mitochondrial Related Disease Research Center, Hualien Tzu Chi Hospital, Buddhist Tzu Chi Medical Foundation, Hualien 970, Taiwan, ROC; 6 Department of Medical Research, China Medical University Hospital, China Medical University, Taichung 404, Taiwan, ROC; 7 Graduate Institute of Biomedical Sciences, China Medical University, Taichung 404, Taiwan, ROC; 8 College of Physical Education, Chengdu University, Sichuan, China

**Keywords:** DNA repair, inflammation, p16^Ink4a^, γ-H2AX, CD11b, HIIT, cycling

## Abstract

p16^INK4a^ expression is a robust biomarker of senescence for stem cells in human tissues. Here we examined the effect of exercise intensity on *in vivo* senescence in skeletal muscle, using a randomized counter-balanced crossover design. Biopsied vastus lateralis of 9 sedentary men (age 26.1 ± 2.5 y) were assessed before and after a single bout of moderate steady state exercise (SSE, 60% maximal aerobic power) and high intensity interval exercise (HIIE, 120% maximal aerobic power) on a cycloergometer accumulating same amount of cycling work (in kilojoule). Increases in cell infiltration (+1.2 folds), DNA strand break (+1.3 folds), and γ-H2AX^+^ myofibers (+1.1 folds) occurred immediately after HIIE and returned to baseline in 24 h (*p* < 0.05). Muscle p16^Ink4a^ mRNA decreased 24 h after HIIE (−57%, *p* < 0.05). SSE had no effect on cell infiltration, p16^Ink4a^ mRNA, and DNA strand break in muscle tissues. Senescence-lowering effect of HIIE was particularly prominent in the muscle with high pre-exercise p16INK4a expression, suggesting that exercise intensity determines the level of selection pressure to tissue stem cells at late senescent stage in human skeletal muscle. This evidence provides an explanation for the discrepancy between destructive nature of high intensity exercise and its anti-aging benefits.

## INTRODUCTION

The majority of cells in human body have short lifespans [1]. Therefore, senescent cells are widely detectable in tissues during embryonic stage [2] and young adulthood [3]. A dynamic balance between cell regeneration and death is required to maintain youth levels and total cell number of a tissue [4]. Tissue renewal requires infiltration of stem cells from bone marrow into peripheral tissues to replenish local progenitors [5]. These replicable cells express a wide spectrum of cyclin-dependent kinase inhibitor p16^INK4a^. The amount of p16^INK4a^ transcripts is a commonly used indicative for senescence of replicable cells, validated by increasing its mRNA levels with human ages [6, 7]. p16^INK4a^ expression in stem cells elevates progressively following a limited cycle of cell divisions [8, 9]. Stem cells with increased p16^INK4a^ expression lose their ability to regenerate, occupy cellular niches, and increase tissue inflammation [10]. They are closely associated with decreased physical fitness and delayed recovery against injurious challenges [11, 12].

The amount of p16^INK4a^ expression seems to associate with accumulation of DNA damage [13]. To maintain genetic stability for normal cell divisions, DNA strand break immediately induces DNA repair mechanism by increasing phosphorylation of histone H2AX (γ-H2AX) [14]. γ-H2AX attracts proteins participating DNA repair process for double strand breaks [15]. The effects of an acute bout of exercise on the levels of γ-H2AX and DNA fragmentation have been rarely reported in human skeletal muscle.

We have previously shown a decreased p16^INK4a^ mRNA 1 day after an acute bout of resistance exercise in human skeletal muscle [3]. Resistance exercise is generally known to induce significant muscle damage and inflammation [16]. However, cellular senescence marker does not seem to change after aerobic exercise at 70% ${\dot{\text{V}}\text{O}}_{\text{2peak}}$ [17, 18]. In this study, we asked the question whether the cellular senescence-lowering effect of exercise in human skeletal muscle can occur only at the intensity sufficient to induce DNA damage and inflammation. To control the potential influence of exercise volume, p16^INK4a^ mRNA and γ-H2AX foci in human skeletal muscle were assessed after an acute bout of HIIE and SSE accumulating same amount of cycling work (in kilojoules).

## RESULTS

**Figure 1:**
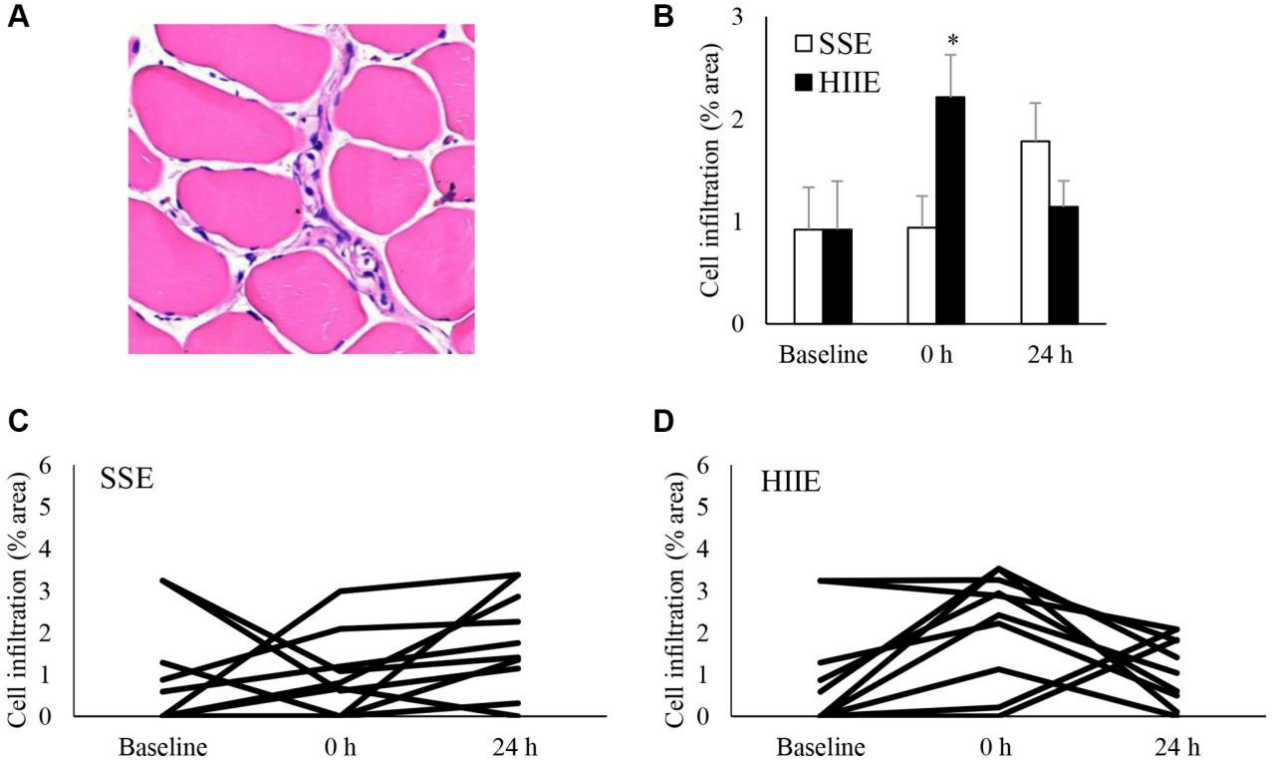
**Cell infiltration in human skeletal muscle after SSE and HIIE.** A representative graph of cell infiltration increased immediately following HIIE (**A**). This response is delayed after SSE with similar amount of cycling work (**B**). Individual response after SSE (**C**) and HIIE (**D**) are shown in the lower panel. ^*^Significant difference against pre-exercise baseline, *p* < 0.05. Abbreviations: SSE: steady state cycling exercise (60% maximal aerobic power); HIIE: high intensity interval cycling exercise (120% maximal aerobic power).

Cell infiltration is a hallmark of muscle inflammation after injuries. Figure 1 shows the amount of cell infiltration in human skeletal muscle following an acute bout of SSE or HIIE. A representative hematoxylin and eosin stain (HE) stains of muscle cross-section is shown in Figure 1A. At pre-exercise baseline, approximately 1% area of nucleated cell aggregation in collapsed and shrinking myofibers was observed for the entire muscle cross-section among the young sedentary participants. HIIE doubled the amount of cell infiltration (*d* = 1.00; *p* < 0.05) in skeletal muscle immediately after exercise and returned to baseline within 24 h (Figure 1B). SSE produced a minimal response of cell infiltration 24 h after exercise (not significant), particularly for those participants with low pre-exercise cell infiltration. Individual responses of cell infiltration against SSE and HIIE are shown in Figure 1C and 1D, respectively.

**Figure 2:**
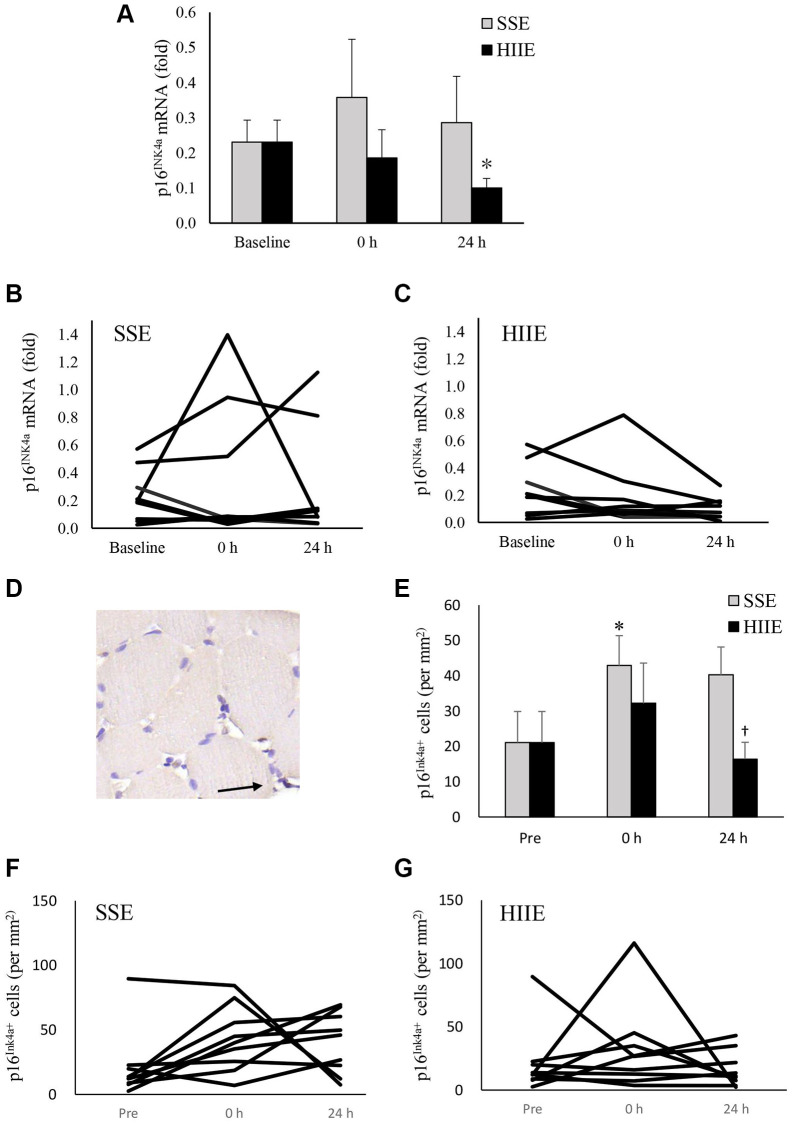
**p16^INK4a^ mRNA in human skeletal muscle 24 h after SSE and HIIE.** The cellular senescence marker p16^INK4a^ mRNA decreased 24 h after HIIE (*d* = 0.90, *p* = 0.04), whilst no change was observed after SSE at similar amount of cycling work (**A**). Individual responses in p16^INK4a^ mRNA to SSE and HIIE are shown in (**B** and **C**), respectively. p16^INK4a^-expressing cells (representing all replicable cells) were located outsides myofibers, indicated by an arrow to brown precipitates (**D**). p16^INK4a^-expression cells (replicable cells) increased immediately after HIIE and returned to baseline in 24 h (**E**). Individual responses to SSE and HIIE are shown in (**F** and **G**), respectively. ^*^Significant difference against pre-exercise baseline, *p* < 0.05. ^†^Significance against SSE, *p* < 0.05. Abbreviations: SSE: steady state cycling exercise (60% maximal aerobic power); HIIE: high intensity interval cycling exercise (120% maximal aerobic power).

In this study, p16^INK4a^ mRNA was used to indicate the levels of cellular senescence in human skeletal muscle (Figure 2A–2C). HIIE decreased p16^INK4a^ mRNA in muscle tissues following a 24-h recovery (*d* = 0.90, *p* = 0.04), whereas SSE showed no effect on p16^INK4a^ mRNA in muscle tissues (Figure 2A). The individual responses of p16^INK4a^ mRNA to HIIE and SSE are shown in Figure 2B and 2C. Data from IHC stains of muscle cross-section was used to identify the location and the amount of p16^INK4a^-expressing cells (exemplary in Figure 2D), which represents the number of all replicable cells regardless intensity of p16^INK4a^ positive signals. There is trend of increases in p16^INK4a+^ cell number immediately after both SSE and HIIE (Figure 2E). A significant difference between SSE and HIIE was found 24 h after exercise (*d* = 1.90, *p* < 0.01). The individual responses of p16^INK4a+^ cell numbers in muscle tissues among these sedentary participants after SSE and HIIE are shown in Figure 2F and 2G.

**Figure 3:**
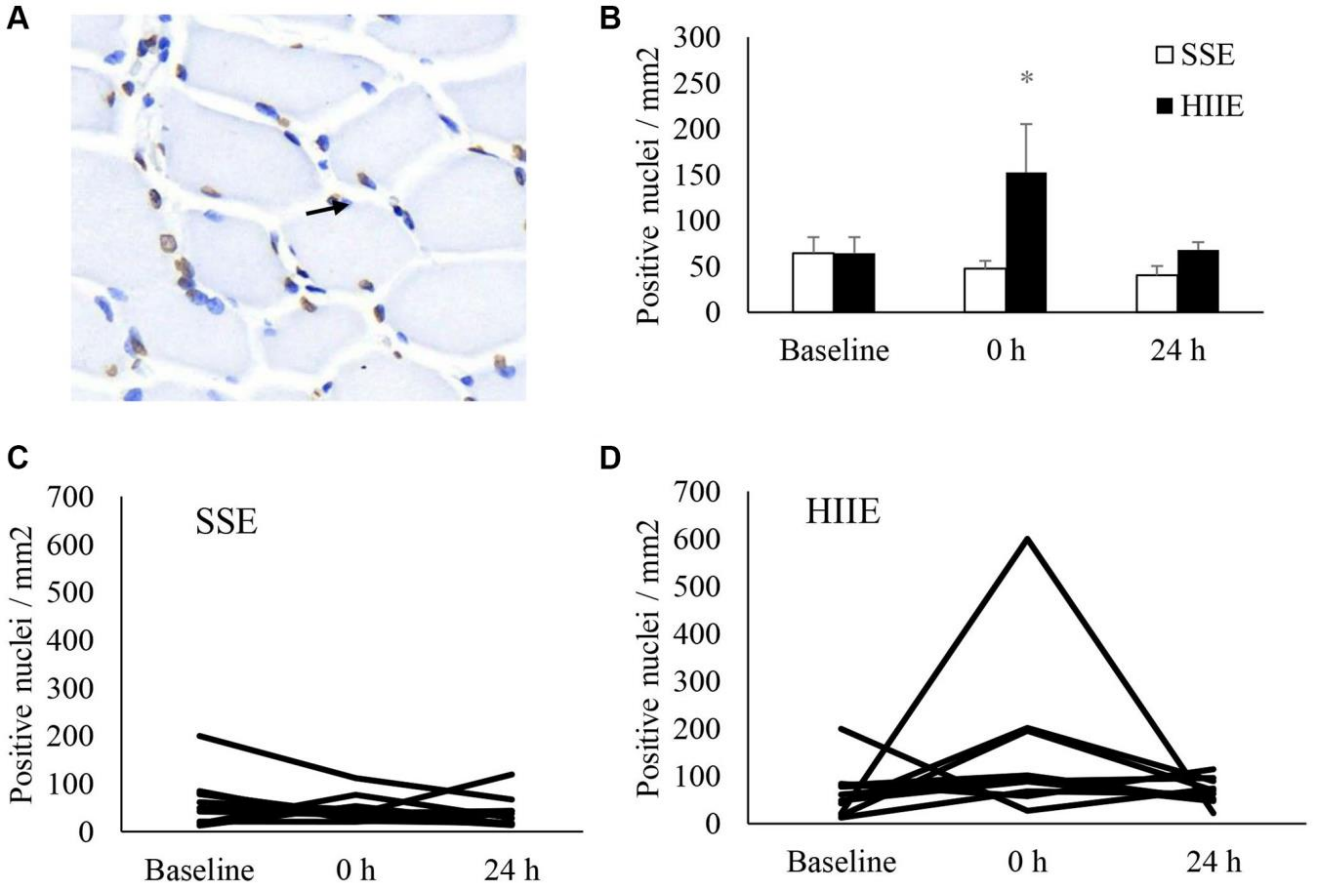
**DNA strand break in human skeletal muscle after SSE and HIIE.** Approximately one third of nuclei (blue) showed fragmented DNA (brown) at pre-exercise baseline (**A**). The fragmented DNA elevated by 1.3 folds immediately after HIIE and returned to baseline in 24 h (**B**). Individual response after SSE (**C**) and HIIE (**D**) are shown in the lower panel. ^*^Significance against pre-exercise baseline, *p* < 0.05. ^†^Significant difference against SSE, *p* < 0.05. Abbreviations: SSE: steady state cycling exercise (60% maximal aerobic power); HIIE: high intensity interval cycling exercise (120% maximal aerobic power).

Figure 3 represents DNA strand break (TUNEL assay) of muscle cross-section after HIIE. Before exercise, fragmented DNA was detected in both morphologically normal myofibers and the nucleated cells outside myofibers at a roughly similar proportion (Figure 3A), indicating a dynamic balance of DNA strand break and repair in normal nuclei of human skeletal muscle. Increased amount of these fragmented DNA (+1.3-fold, *d* = 0.77, *p* < 0.05) was observed immediately after HIIE and returned to baseline in 24 h (Figure 3B). The individual responses of DNA fragmentation to HIIE and SSE trials are shown in Figure 3C and 3D.

**Figure 4:**
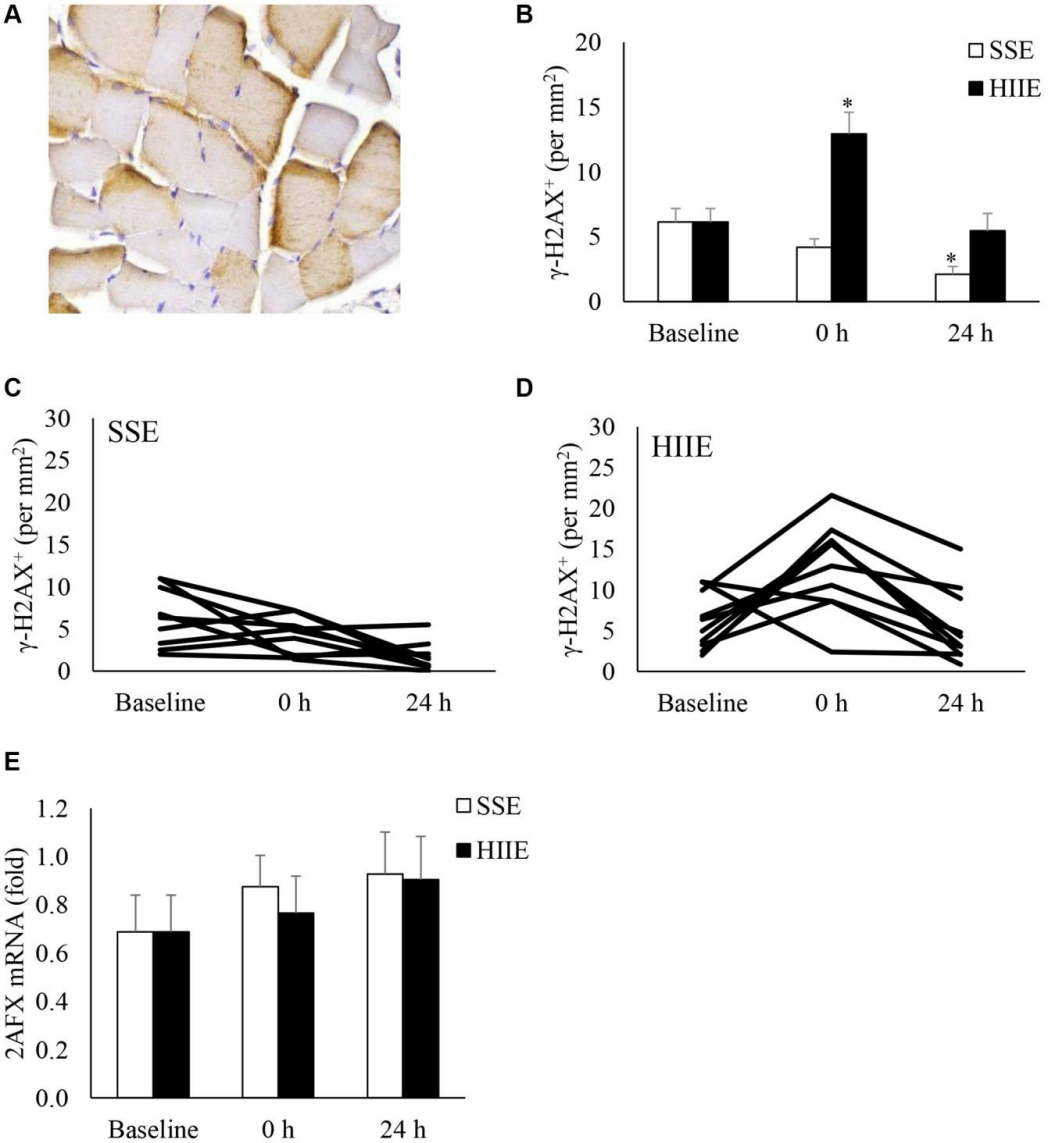
**γ-H2AX^+^ myofibers in human skeletal muscle after SSE and HIIE.** A representative muscle cross-section of immunohistochemical stain indicates brown γ-H2AX signals concentrated near periphery of myofibers (**A**). γ-H2AX^+^ myofibers elevated immediately only after HIIE (not SSE) and returned to baseline in 24 h (**B**). Individual responses to SSE and HIIE are shown in (**C** and **D**), respectively. H2AX mRNA was not influenced by exercise (**E**). ^*^Significant difference against pre-exercise baseline, *p* < 0.05; ^†^Significance against SSE, *p* < 0.05. Abbreviations: SSE: steady state cycling exercise (60% maximal aerobic power); HIIE: high intensity interval cycling exercise (120% maximal aerobic power).

Figure 4 shown γ-H2AX (a phosphorylated histone H2AX on Ser-139) of muscle cross-section after exercise. γ-H2AX is more concentrated near the edge of cytoplasm in myofibers (Figure 4A). γ-H2AX^+^ myofibers were unaltered after SSE (Figure 4B). HIIE increased γ-H2AX^+^ myofiber immediately after HIIE (+ 1.1-fold, *d* = 1.55, *p* < 0.05) and returned to pre-exercise baseline in 24 h. Despite a decreased γ-H2AX^+^ myofibers 24 h following SSE (−66%, *d* = 0.69, *p* = 0.09), no statistical significance between HIIE and SSE was reached 24 h after exercise. The individual responses of γ-H2AX^+^ cells in SSE and HIIE trials are shown in Figure 4C and 4D. No change in H2AX mRNA was observed after both SSE and HIIE (Figure 4E).

## DISCUSSION

The intensity effects of aerobic exercise on cellular senescence and DNA repair response of human skeletal muscle have not been previously documented. In this study, the major findings are as follows: (1) Cellular senescence-lowering effect of aerobic exercise can occur only at high intensity. SSE with similar exercise work failed to lower the p16^INK4a^ mRNA in human skeletal muscle within the 24-h recovery period; (2) HIIE triggered immediate increases in cell infiltration and γ-H2AX^+^ myofibers, followed by a decreased p16^INK4a^ mRNA in human skeletal muscle 24 h after recovery; (3) By further examining the individual responses, the senolytic effect of HIIE were contributed solely from those participants with high pre-exercise p16^INK4a^ mRNA in skeletal muscles. High intensity exercise is known to cause greater levels of lactate production and acidosis than low intensity exercise [19]. Decreased pH has been reported to be a danger signal to activate innate immune response [20] and immune cells are functioned to recognize and clear senescent cells by phagocytosis in humans [21, 22]. Therefore, the acute changes in the microenvironment during and after exercise may be a selection pressure to aged stem cell population resided in the skeletal muscle. Exercise intensity determines the magnitude of cell renewal during a brief period of inflammation.

Decreased p16^INK4a^ mRNA without significant changes in p16^INK4a^-expressing cells 24 h following HIIE suggest a shift in senescent profile of stem cell population in challenged muscle tissues. Stem cells express a wide spectrum of p16^INK4a^ mRNA, and its level increments with the number of cell divisions [23–25]. One study reported a correlation between p16^INK4a^ mRNA in skeletal muscle and human age [17]. However, the number of p16^INK4a^-expressing cells in human skeletal muscle were similar between young and old men [26]. p16^INK4a+^ cells of muscle cross-section observed in the study may reflect, to some extent, the amount of bone marrow derived stem cells and progenitors (or all replicable cells) homed to the challenged muscle tissues for renewal [27].

Muscle inflammation, characterized by cell infiltration in injured tissues, is required for muscle regeneration, mediated by a complex muscle-myeloid cell interaction [28]. Here, we demonstrated that the amount of cell infiltration into human skeletal muscle is intensity dependent and is likely associated with an acute release of bone marrow cells into circulation [29]. Bone marrow stem cells develops to phagocytes and multipotent stem cells to eliminate senescent cells and replenish local stem cells in challenged skeletal muscle [30]. Taken together, decreased cellular senescence 24 h after HIIE is best explained by senescent cell clearance following a brief increase of bone marrow cell infiltration into challenged skeletal muscle to participate in the early phagocytic and late regenerative phases of inflammation [28, 29].

DNA damage is a potent stimulator of inflammation [31]. In this study, a fast resolution of DNA damage/repair response 24 h following HIIE suggests an efficient clearance of senescent cells with DNA damage in human skeletal muscle to resolve the inflammation. In the study, we have shown a transitory increase in DNA strand break and γ-H2AX^+^ cells in human skeletal muscle immediately after HIIE. Increasing *γ*H2AX attracts proteins for DNA repair to the sites of double strand break in the nucleosomes [32, 33]. This acute response of increased *γ*H2AX fits well with a diminished DNA strand break 24 h after HIIE, providing *in vivo* evidence of DNA repair in exercised human skeletal muscle. We have previous reported a reversed DNA fragmentation to pre-exercise level in 3 h following a cycling exercise at the aerobic power equivalent to 70% ${\dot{\text{V}}\text{O}}_{\text{2peak}}$ [18]. It remains to be examined whether the outcomes of DNA repair are associated with *in situ* excision repair or nucleus replacement from surrounding satellite cells outside myofibers.

The anti-aging effect of exercise has been supported by a significant reduction in all-cause mortality to the similar level as observed among individuals with high physical activity after increasing physical activity from low/moderate to vigorous levels during middle age [34]. However, increasing exercise intensity is apparently more destructive to skeletal muscle than moderate intensity despite a similar total work accomplished. The results of the study provide evidence to explain the discrepancy between destructive nature of high intensity exercise and its anti-aging benefits. Bone marrow cell infiltration into skeletal muscle is required to induce a greater scale of tissue remodeling to lower age profile of stem cells, which is more prominent using high intensity exercise.

The senescence lowering outcomes observed in the present study may also explain the superiority of high intensity exercise in increasing insulin sensitivity or anabolic potential of skeletal muscle than moderate intensity exercise [35]. It has been shown significant improvements in muscle mass gain in octogenarians after 4-week of high intensity interval training (100−110% maximal aerobic power, 90-sec cycling for 12 sessions) [36]. SSE (70% heart rate reserve, 45-min treadmill running, 24 weeks) has no effect in lean mass gain [37]. Muscle tissue containing less senescent cells is expected to confer better growth potential of extracting substrates (glucose, fatty acids, and amino acids) for reconstruction than the muscle tissue containing more senescent cells. Senescent cells are known to release a variety of proinflammatory molecules, collectively termed as the senescence-associated secretory phenotype (SASP), which attracts immune clearance [38]. Physically inactive individuals may accumulate more senescent cells in peripheral tissues and dilute the bone marrow resource for tissue renewal due to more competition among aged tissues when bone-to-weight ratio increases during growth [4].

A major limitation of the study is the follow-up time after exercise. We failed to find any significant effect of SSE on cellular senescence biomarkers during the 24 h recovery period. However, we were uncertain whether SSE has delayed response on cellular senescence in muscle after 24 h. Another limitation is measurement of p16^INK4a^ protein using IHC cannot be used to indicate the levels of protein expression. In our study, IHC was employed to represent p16^INK4a^-expressing cell number instead of protein levels. p16^INK4a^ protein measurement is required to confirm evidence to generate knowledge.

### Practical application

This study provides evidence which indicates senolytic effect of a very short duration of exercise (10 min including rest intervals). However, no evidence of such senolytic effect was observed on SSE with similar accumulated work in kilojoules. Our results highlight the importance of exercise intensity for its anti-aging benefit. The cell infiltration outcome after HIIE points to a possibility that the senolytic effect is mediated by muscle inflammation. Inflammation inevitably brings a short period of discomfort during and after exercise. Therefore, developing interventions to resolve muscle inflammation at a faster pace may be a future direction to optimize the senolytic effect of exercise in practice.

## METHODS

### Participants

This study was approved by the Institutional Review Board of University of Taipei (Approved number IRB-2018-078) and was conformed to the spirit of Declaration of Helsinki. A total of fifteen young men voluntarily enrolled this study with six dropouts associated with time conflict. Nine sedentary men (age 26.1 ± 2.5 y) completed the study. Inclusion criteria were sedentary men (exercise < 1 day per week) who were capable to conduct cycling exercise. Exclusion criteria were users of medication and cigarette. Baseline characteristics of the participants are shown in Table 1. All participants were informed the study purpose, potential risks, and procedures before beginning of the study.

**Table 1:** Characteristics of participants.

	**Mean ± SE**
Number of participants	9
Age (y)	26.1 ± 2.5
Height (cm)	171.0 ± 6.7
Weight (kg)	67.2 ± 8.3
BMI	23.0 ± 2.3
VO_2peak_ (ml/min/kg)	33.6 ± 4.1
Maximal aerobic power (watt)	174.1 ± 25.5

### Study design

A counter-balanced crossover design was performed to compare the effects of two exercise intensities (SSE: 60% maximal aerobic power; HIIE: 120% maximal aerobic power), accumulating the same work on a cycloergometer. The primary outcome variables were p16^INK4a^ expression and γ-H2AX^+^ myofibers in skeletal muscle. Baseline muscle biopsy to vastus lateralis muscle was performed 3 weeks prior to the study. One week after the baseline muscle biopsy, participants familiarized with the experimental procedures and lab equipment. Aerobic power at maximal oxygen uptake (maximal aerobic power) of each participant was measured 1 week before exercise challenge. On the day of exercise challenge, half participants performed HIIE and the rest of participants performed SSE for 10 min. In the crossover trial, participants performed the same amount of work on alternative intensity (HIIE switched to SSE, or vice versa). Total work on the cycloergometer was recorded during the first cycling trial to match the actual work performed between HIIE and SSE. The crossover trials were separated for > 3 weeks. Post-exercise muscle samples were collected immediately after (0 h) and 24 h after HIIE and SSE. To minimize potential nutritional influence on exercise intervention, a cane of Ensure (Abbott Nutrition, Taipei, Taiwan) was provided 12 h (2200) and 2 h (0800) before exercise. Participants were asked not to alter their dietary habit and allowed to drink water *ad libitum* before the study and during the post-exercise recovery.

### Maximal aerobic power

To obtain maximal aerobic power (cycling power at ${\dot{\text{V}}\text{O}}_{\text{2peak}}$) of each participant, a graded exercise-to-exhaustion test protocol was performed on an electronically braked cycloergometer (Monark LC6, Stockholm, Sweden) after a familiarization trial. ${\dot{\text{V}}\text{O}}_{\text{2}}$ and ${\dot{\text{V}}\text{CO}}_{\text{2}}$ were measured by a gas analyzer (Cortex Metalyzer 3B, Leipzig, Germany). The initial workload started from 50 W and incrementally elevated 20 W every 3 min until volitional exhaustion. Criteria to obtain ${\dot{\text{V}}\text{O}}_{\text{2peak}}$ required to achieve two of the following four conditions: (1) failed to maintain 60 rpm for > 10 sec; (2) RER ≥ 1.2; (3) heart rate > 95% HR_max_; (4) RPE > 9. We used the rating of the perceived exertion (RPE) scaled from 0 to 10 to monitor the difficulty of the exercise at the end of each exercise challenge [39].

### Exercise challenges

Exercise procedure for SSE and HIIE was controlled on a computer with installed Monark Test Software. At the beginning of the challenges, each participant completed a 3-min warm-up at 50 watts on a cycloergometer. For SSE, each participant cycled at the work rate of 60% power at ${\dot{\text{V}}\text{O}}_{\text{2peak}}$ to maintained above 70 rpm for a total period of ~10 min. For HIIE, each participant cycled at the work rate of 120% maximal aerobic power to maintain above 70 rpm for 20 s with a 20-s rest interval up to 15 times (~10 min). To avoid the effect of inertia, participants were instructed to increase the number of rpm 3–5 s before finishing each rest phase [40]. Two exercise challenges were controlled to achieve the same amount of total work during their crossover trial.

### Muscle biopsy

Muscle biopsy was conducted by a physician under local anesthesia (2% lidocaine) using a 18-G Temno disposable cutting needle (Cardinal Health, McGaw Park, Illinois, USA) inserted into the vastus lateralis according to a previously published procedure [41]. The first muscle tissue collection (pre-exercise baseline) was performed on one leg, 3 weeks before exercise challenge. The second and third muscle biopsies were performed immediately after (0 h) and 24 h after SSE or HIIE during each trial. To prevent the interference of micro lesion on inflammatory response caused by previous needle biopsy, the next muscle biopsy was always conducted on the contralateral leg. One muscle sample from the first puncture was placed into liquid nitrogen for real-time PCR analysis and another piece of muscle sample from the second puncture was placed in a conical vial containing 4% paraformaldehyde solution (PFA) for immunohistochemical analysis (IHC).

### Muscle staining

Paraffin-embedded muscle tissues were cross-sectioned 3 h after the biopsy for HE staining and IHC staining according to the previously established protocol [3]. Serial muscle cross-sections (3 μm thick) were used to detect various biomarkers in muscle tissues. HE staining and IHC staining of muscle cross-section were performed by pathologists from Toson Technology Corporation (Zhubei City, Hsinchu, Taiwan).

Muscle necrosis (cell infiltration in collapse or shrinking myofibers) in muscle tissue was assessed by HE staining (TA01HE, BioTnA, Kaohsiung, Taiwan) according to previously published methods [42]. We modified the procedure by including an additional criteria of nucleated cell aggregation > 7 in the disrupted or shrinking myofibers of muscle cross-sections. Muscle tissues were sliced (2–3 μm thick) from the paraffinize blocks, de-paraffinized in xylene and rehydrated in a graded alcohol series using ethanol (99.9%, 95%, 85% and 75%) each for 2 min, and stained with hematoxylin (TA01NB, BioTnA, Kaohsiung, Taiwan) for 3 min. After being washed in distilled water and muscle tissue sections were stained with eosin for 15 s and dehydrated in ethanol.

IHC stains of muscle cross-section were labelled using primary antibodies against p16^Ink4a^ and γ-H2AX for labelling. Fragmented DNA (DNA damage marker) was assessed by Terminal deoxynucleotidyl transferase (TdT) dUTP Nick-End Labeling (TUNEL) assay using the BioTnA TdT *In Situ* Apoptosis Detection Kit (TAAP01D, Torson, Hsinchu, Taiwan) according to manufacturer’s instruction. The primary antibody that recognizes p16^Ink4a+^ cells was anti-human p16^Ink4a^ antibody (1:100, Abcam, ab108349, Boston, MA, USA). Incubation time of muscle cross-section with primary anti-body was 16 h. p16^INK4a-^expressing cells (p16^INK4a+^ cells) were counted based on total number per area (not signal intensity) of muscle cross-sections. The criteria to determine p16^INK4a+^ cells included (a) intact and whole cell; (b) both brown and blue precipitates stained on the entire cell nuclei; (c) cell nuclei must contact near the muscle fiber. The primary antibody that recognizes γ-H2AX to assess DNA repair response after damage was anti-phospho-S193 antibody (1:400, Abcam, ab2893, Boston, MA, USA). The positive γ-H2AX^+^ cells were counted according to the criteria modified from the previous studies [43, 44]: (a) brown signals covering 90% of myofiber; (b) Scoring: dark brown myofibers multiplied by 2, light brown myofibers multiplied by 1.

Whole-slide images were viewed using DSAssistant and EasyScanner software at Toson Technology Corporation (Zhubei City, Hsinchu, Taiwan). Glass slides containing all biopsy time points of each participant were positioned in paralleled and were digitized using multi-level panoramic scanning of pathological slices with a Motic Easyscan Digital Slide Scanner (Motic Hong Kong Limited, Hong Kong, China) at 40 X (0.26 μm/pixel). Cells presenting specific protein markers on IHC stains were quantified and expressed as positive signal number per field area (mm^2^) regardless their signal intensity. The positive signal counting should be regarded as the number of cells that expresses the protein of interest. Stained cross-sections were viewed at a magnification to 40 X. To obtain unbiased IHC counting value, two independent assessors counted positive signals of the same image using the same counting criteria. IHC outcomes were accepted only when intraclass correlation efficient (ICC) of the counting between two assessors was higher than 0.7.

### RNA analysis

RNA extraction from muscle samples used RNeasy kit (QIAGEN 74104, Germantown, MD, USA), RNase-Free DNase Set (QIAGEN 79254, Germantown, MD, USA), and Proteinase K (QIAGEN 19131, Germantown, MD, USA) following the muscle homogenization. The frozen muscle sample (5–10 mg) was transferred into a 1.5 ml tube containing 600 μl of β-mercaptoethanol (β-ME) with RLT buffer on ice. They were homogenized immediately using Polytron (model PT 3000D, Brinkmann, Zurich, Switzerland) for 30 sec at 9000 rpm (50% of the maximum speed). Extracted RNA (1 μg in 20 μl) was reversely transcribed to cDNA using iScript cDNA Synthesis Kit (Bio-Rad 170-8890, Hercules, CA, USA).

Real-time PCR was performed using MyiQ Single Color Real-Time PCR Detection System (Bio-Rad, Hercules, CA, USA), TaqMan Probe (Sigma-Aldrich, Singapore) and iQ Supermix kit (Bio-Rad 170-8860, Hercules, CA, USA). The PCR conditions for all genes consisted of one denaturing cycle at 90°C for 30 s, annealing at 60°C for 60 s and elongation at 72°C for 60 s. The PCR samples were subjected to a melting curve analysis. The cycling parameters were 95°C for 3 min, then 50 cycles at 95°C for 10 s and 58°C for 30 s. Gene expression, normalized to the geometric mean of a housekeeping genes (RPP30), was quantified using the 2−(ΔCt) method and expressed as fold difference relative to the RPP30. The primers and probes used to amplify the target were supplied from Bio-Rad PrimePCR™ Probe Assay according to Unique Assay ID as follows: p16^INK4a^ (or CDKN2A), qHsaCEP0057827; H2AX, qHsaCEP0055238; and internal standard RPP30, qHsaCEP0052683.

### Statistical analysis

All data are expressed as means ± standard error (SE). The data were analyzed using repeated measure ANOVA using a software (SPSS 25.0, IBM, NY, USA). Effect size was indicated by Cohen’s *d*. When *d* values between 0.5 to 0.8 were considered medium effect, and above 0.8 were considered large effect. Paired *t*-test was used to compare mean between SSE and HIIE. Type 1 error of *p* < 0.05 for comparison between means was considered significant. The magnitude of the correlations was identified as follows: trivial (0.00–0.09), small (0.10–0.29), moderate (0.30–0.49), large (0.50–0.69), very large (0.70–0.89), nearly perfect (0.90–0.99), and perfect (1.00).

## References

[1] Spalding KL, Bhardwaj RD, Buchholz BA, Druid H, Frisén J. Retrospective birth dating of cells in humans. Cell. 2005; 122:133–43. 10.1016/j.cell.2005.04.028 16009139

[2] Storer M, Mas A, Robert-Moreno A, Pecoraro M, Ortells MC, Di Giacomo V, Yosef R, Pilpel N, Krizhanovsky V, Sharpe J, Keyes WM. Senescence is a developmental mechanism that contributes to embryonic growth and patterning. Cell. 2013; 155:1119–30. 10.1016/j.cell.2013.10.041 24238961

[3] Lee TXY, Wu J, Jean WH, Condello G, Alkhatib A, Hsieh CC, Hsieh YW, Huang CY, Kuo CH. Reduced stem cell aging in exercised human skeletal muscle is enhanced by ginsenoside Rg1. Aging (Albany NY). 2021; 13:16567–76. 10.18632/aging.203176 34181580 PMC8266347

[4] Lin YH, Tsai SC, Chuang SJ, Harris MB, Masodsai K, Chen PN, Hsieh CC, Killian T, Huang CY, Kuo CH. Whole-life body composition trajectory and longevity: role of insulin. Aging (Albany NY). 2021; 13:9719–31. 10.18632/aging.202727 33744845 PMC8064149

[5] Dewi L, Lin YC, Nicholls A, Condello G, Huang CY, Kuo CH. Pax7^+^ Satellite Cells in Human Skeletal Muscle After Exercise: A Systematic Review and Meta-analysis. Sports Med. 2023; 53:457–80. 10.1007/s40279-022-01767-z 36266373

[6] Liu Y, Sanoff HK, Cho H, Burd CE, Torrice C, Ibrahim JG, Thomas NE, Sharpless NE. Expression of p16(INK4a) in peripheral blood T-cells is a biomarker of human aging. Aging Cell. 2009; 8:439–48. 10.1111/j.1474-9726.2009.00489.x 19485966 PMC2752333

[7] Ressler S, Bartkova J, Niederegger H, Bartek J, Scharffetter-Kochanek K, Jansen-Dürr P, Wlaschek M. p16INK4A is a robust in vivo biomarker of cellular aging in human skin. Aging Cell. 2006; 5:379–89. 10.1111/j.1474-9726.2006.00231.x 16911562

[8] Hayflick L. Biological aging is no longer an unsolved problem. Ann N Y Acad Sci. 2007; 1100:1–13. 10.1196/annals.1395.001 17460161

[9] Chen JH, Hales CN, Ozanne SE. DNA damage, cellular senescence and organismal ageing: causal or correlative? Nucleic Acids Res. 2007; 35:7417–28. 10.1093/nar/gkm681 17913751 PMC2190714

[10] He S, Sharpless NE. Senescence in Health and Disease. Cell. 2017; 169:1000–11. 10.1016/j.cell.2017.05.015 28575665 PMC5643029

[11] Baker DJ, Childs BG, Durik M, Wijers ME, Sieben CJ, Zhong J, Saltness RA, Jeganathan KB, Verzosa GC, Pezeshki A, Khazaie K, Miller JD, van Deursen JM. Naturally occurring p16(Ink4a)-positive cells shorten healthy lifespan. Nature. 2016; 530:184–9. 10.1038/nature16932 26840489 PMC4845101

[12] Wilkinson HN, Hardman MJ. Senescence in Wound Repair: Emerging Strategies to Target Chronic Healing Wounds. Front Cell Dev Biol. 2020; 8:773. 10.3389/fcell.2020.00773 32850866 PMC7431694

[13] Demaria M, O'Leary MN, Chang J, Shao L, Liu S, Alimirah F, Koenig K, Le C, Mitin N, Deal AM, Alston S, Academia EC, Kilmarx S, et al. Cellular Senescence Promotes Adverse Effects of Chemotherapy and Cancer Relapse. Cancer Discov. 2017; 7:165–76. 10.1158/2159-8290.CD-16-0241 27979832 PMC5296251

[14] Kuo LJ, Yang LX. Gamma-H2AX - a novel biomarker for DNA double-strand breaks. In Vivo. 2008; 22:305–9. 18610740

[15] d'Adda di Fagagna F. Living on a break: cellular senescence as a DNA-damage response. Nat Rev Cancer. 2008; 8:512–22. 10.1038/nrc2440 18574463

[16] Noakes TD. Effect of exercise on serum enzyme activities in humans. Sports Med. 1987; 4:245–67. 10.2165/00007256-198704040-00003 3306866

[17] Balan E, De Groote E, Bouillon M, Viceconte N, Mahieu M, Naslain D, Nielens H, Decottignies A, Deldicque L. No effect of the endurance training status on senescence despite reduced inflammation in skeletal muscle of older individuals. Am J Physiol Endocrinol Metab. 2020; 319:E447–54. 10.1152/ajpendo.00149.2020 32691630

[18] Wu J, Saovieng S, Cheng IS, Liu T, Hong S, Lin CY, Su IC, Huang CY, Kuo CH. Ginsenoside Rg1 supplementation clears senescence-associated β-galactosidase in exercising human skeletal muscle. J Ginseng Res. 2019; 43:580–8. 10.1016/j.jgr.2018.06.002 31695564 PMC6823780

[19] Robergs RA, Ghiasvand F, Parker D. Biochemistry of exercise-induced metabolic acidosis. Am J Physiol Regul Integr Comp Physiol. 2004; 287:R502–16. 10.1152/ajpregu.00114.2004 15308499

[20] Rajamäki K, Nordström T, Nurmi K, Åkerman KE, Kovanen PT, Öörni K, Eklund KK. Extracellular acidosis is a novel danger signal alerting innate immunity via the NLRP3 inflammasome. J Biol Chem. 2013; 288:13410–9. 10.1074/jbc.M112.426254 23530046 PMC3650379

[21] Kay MM. Mechanism of removal of senescent cells by human macrophages in situ. Proc Natl Acad Sci U S A. 1975; 72:3521–5. 10.1073/pnas.72.9.3521 1059140 PMC433027

[22] Prata LGP, Ovsyannikova IG, Tchkonia T, Kirkland JL. Senescent cell clearance by the immune system: Emerging therapeutic opportunities. Semin Immunol. 2018; 40:101275. 10.1016/j.smim.2019.04.003 31088710 PMC7061456

[23] Campisi J. Aging, cellular senescence, and cancer. Annu Rev Physiol. 2013; 75:685–705. 10.1146/annurev-physiol-030212-183653 23140366 PMC4166529

[24] Hayflick L. The Limited In Vitro Lifetime of Human Diploid Cell Strains. Exp Cell Res. 1965; 37:614–36. 10.1016/0014-4827(65)90211-9 14315085

[25] Kim H, You S, Farris J, Kong BW, Christman SA, Foster LK, Foster DN. Expression profiles of p53-, p16(INK4a)-, and telomere-regulating genes in replicative senescent primary human, mouse, and chicken fibroblast cells. Exp Cell Res. 2002; 272:199–208. 10.1006/excr.2001.5420 11777345

[26] Dungan CM, Peck BD, Walton RG, Huang Z, Bamman MM, Kern PA, Peterson CA. In vivo analysis of γH2AX+ cells in skeletal muscle from aged and obese humans. FASEB J. 2020; 34:7018–35. 10.1096/fj.202000111RR 32246795 PMC7243467

[27] Chikenji TS, Saito Y, Konari N, Nakano M, Mizue Y, Otani M, Fujimiya M. p16^INK4A^-expressing mesenchymal stromal cells restore the senescence-clearance-regeneration sequence that is impaired in chronic muscle inflammation. EBioMedicine. 2019; 44:86–97. 10.1016/j.ebiom.2019.05.012 31129096 PMC6604166

[28] Tidball JG. Regulation of muscle growth and regeneration by the immune system. Nat Rev Immunol. 2017; 17:165–78. 10.1038/nri.2016.150 28163303 PMC5452982

[29] Baker JM, Nederveen JP, Parise G. Aerobic exercise in humans mobilizes HSCs in an intensity-dependent manner. J Appl Physiol (1985). 2017; 122:182–90. 10.1152/japplphysiol.00696.2016 27881669 PMC5283849

[30] Zhao E, Xu H, Wang L, Kryczek I, Wu K, Hu Y, Wang G, Zou W. Bone marrow and the control of immunity. Cell Mol Immunol. 2012; 9:11–9. 10.1038/cmi.2011.47 22020068 PMC3251706

[31] Cinat D, Coppes RP, Barazzuol L. DNA Damage-Induced Inflammatory Microenvironment and Adult Stem Cell Response. Front Cell Dev Biol. 2021; 9:729136. 10.3389/fcell.2021.729136 34692684 PMC8531638

[32] Taverna SD, Li H, Ruthenburg AJ, Allis CD, Patel DJ. How chromatin-binding modules interpret histone modifications: lessons from professional pocket pickers. Nat Struct Mol Biol. 2007; 14:1025–40. 10.1038/nsmb1338 17984965 PMC4691843

[33] Kouzarides T. Chromatin modifications and their function. Cell. 2007; 128:693–705. 10.1016/j.cell.2007.02.005 17320507

[34] Byberg L, Melhus H, Gedeborg R, Sundström J, Ahlbom A, Zethelius B, Berglund LG, Wolk A, Michaëlsson K. Total mortality after changes in leisure time physical activity in 50 year old men: 35 year follow-up of population based cohort. BMJ. 2009; 338:b688. 10.1136/bmj.b688 19264819 PMC2654773

[35] Sandvei M, Jeppesen PB, Støen L, Litleskare S, Johansen E, Stensrud T, Enoksen E, Hautala A, Martinmäki K, Kinnunen H, Tulppo M, Jensen J. Sprint interval running increases insulin sensitivity in young healthy subjects. Arch Physiol Biochem. 2012; 118:139–47. 10.3109/13813455.2012.677454 22540332

[36] Blackwell JEM, Gharahdaghi N, Brook MS, Watanabe S, Boereboom CL, Doleman B, Lund JN, Wilkinson DJ, Smith K, Atherton PJ, Williams JP, Phillips BE. The physiological impact of high-intensity interval training in octogenarians with comorbidities. J Cachexia Sarcopenia Muscle. 2021; 12:866–79. 10.1002/jcsm.12724 34060253 PMC8350218

[37] Brightwell CR, Markofski MM, Moro T, Fry CS, Porter C, Volpi E, Rasmussen BB. Moderate-intensity aerobic exercise improves skeletal muscle quality in older adults. Transl Sports Med. 2019; 2:109–19. 10.1002/tsm2.70 31123725 PMC6518946

[38] Ritschka B, Storer M, Mas A, Heinzmann F, Ortells MC, Morton JP, Sansom OJ, Zender L, Keyes WM. The senescence-associated secretory phenotype induces cellular plasticity and tissue regeneration. Genes Dev. 2017; 31:172–83. 10.1101/gad.290635.116 28143833 PMC5322731

[39] Turner AN, Bishop C, Marshall G, Read P. How to monitor training load and mode using sRPE. Prof Strength Cond. 2015; 39:15–20.

[40] Viana RB, Naves JPA, de Lira CAB, Coswig VS, Del Vecchio FB, Vieira CA, Gentil P. Defining the number of bouts and oxygen uptake during the "Tabata protocol" performed at different intensities. Physiol Behav. 2018; 189:10–5. 10.1016/j.physbeh.2018.02.045 29486169

[41] Yang C, Jiao Y, Wei B, Yang Z, Wu JF, Jensen J, Jean WH, Huang CY, Kuo CH. Aged cells in human skeletal muscle after resistance exercise. Aging (Albany NY). 2018; 10:1356–65. 10.18632/aging.101472 29953414 PMC6046228

[42] Knight KR, Messina A, Hurley JV, Zhang B, Morrison WA, Stewart AG. Muscle cells become necrotic rather than apoptotic during reperfusion of ischaemic skeletal muscle. Int J Exp Pathol. 1999; 80:169–75. 10.1046/j.1365-2613.1999.00111.x 10469272 PMC2517767

[43] Varvara PV, Karaolanis G, Valavanis C, Stanc G, Tzaida O, Trihia H, Patapis P, Dimitroulis D, Perrea D. gamma-H2AX: A potential biomarker in breast cancer. Tumour Biol. 2019; 41:1010428319878536. 10.1177/1010428319878536 31552812

[44] Wolff AC, Hammond ME, Hicks DG, Dowsett M, McShane LM, Allison KH, Allred DC, Bartlett JM, Bilous M, Fitzgibbons P, Hanna W, Jenkins RB, Mangu PB, et al, and American Society of Clinical Oncology, and College of American Pathologists. Recommendations for human epidermal growth factor receptor 2 testing in breast cancer: American Society of Clinical Oncology/College of American Pathologists clinical practice guideline update. J Clin Oncol. 2013; 31:3997–4013. 10.1200/JCO.2013.50.9984 24101045

